# A SuperLearner Approach to Predict Run-In Selection in Clinical Trials

**DOI:** 10.1155/2022/4306413

**Published:** 2022-09-10

**Authors:** Corrado Lanera, Paola Berchialla, Giulia Lorenzoni, Aslihan Şentürk Acar, Valentina Chiminazzo, Danila Azzolina, Dario Gregori, Ileana Baldi

**Affiliations:** ^1^Unit of Biostatistics, Epidemiology and Public Health, Department of Cardiac, Thoracic, Vascular Sciences, and Public Health, University of Padova, Via Loredan, 18, 35121 Padova, Italy; ^2^Department of Clinical and Biological Sciences, University of Torino, Via Verdi 8, 10124 Torino, Italy; ^3^Department of Actuarial Sciences, Hacettepe University, Ankara, Turkey 06800; ^4^Department of Environmental and Preventive Sciences, University of Ferrara, Via Fossato di Mortara 64B, 44121 Ferrara, Italy

## Abstract

A critical early step in a clinical trial is defining the study sample that appropriately represents the target population from which the sample will be drawn. Envisaging a “run-in” process in study design may accomplish this task; however, the traditional run-in requires additional patients, increasing times, and costs. The possible use of the available a-priori data could skip the run-in period. In this regard, ML (machine learning) techniques, which have recently shown considerable promising usage in clinical research, can be used to construct individual predictions of therapy response probability conditional on patient characteristics. An ensemble model of ML techniques was trained and validated on twin randomized clinical trials to mimic a run-in process within this framework. An ensemble ML model composed of 26 algorithms was trained on the twin clinical trials. SuperLearner (SL) performance for the Verum (Treatment) arm is above 70% sensitivity. The Positive Predictive Value (PPP) achieves a value of 80%. Results show good performance in the direction of being useful in the simulation of the run-in period; the trials conducted in similar settings can train an optimal patient selection algorithm minimizing the run-in time and costs of conduction.

## 1. Introduction

A critical early step in a clinical trial design is to define the study population from which the sample will be drawn, i.e., to identify the target population most likely to derive benefit from the experimental treatment. Envisaging a “run-in” period in a study design may accomplish this task [1].

The “run-in” is a period before randomization whose aim is detecting/excluding subgroups of patients less likely to respond to the therapy [2].

Incorporating such a pre-randomization period in a study design constitutes a desirable enrichment process of a clinical study [3], but it requires additional patients, increasing times, and costs. Instead, using available a-priori data to inform about the potential patients' outcome, conditionally to the therapy received, could skip such a period. In this way, predictions could improve the population segment selection and the consequent enrolment in clinical trials [4].

The ML is a field of science aimed at fit models with excellent predictive accuracy [5]. A unique feature of ML algorithms is their capability to improve their predictive performance through experience [6]. In a clinical setting, all the amount of different historical and a-priori known information, as well as patient everyday-life data [7], can be employed to learn by improving complex tasks such as classification (e.g., response to therapy and cancer clinical-type classification) and clustering (e.g., identification of groups of patients with shared characteristics).

For these reasons, ML, which is increasingly applied to clinical studies [8, 9], represents a new approach to conducting medical research and developing ways to predict individual outcomes [7–9].

ML techniques have recently been proposed in the design phases of clinical trials, specifically as a method to enforce patient selection in the spirit of a population enrichment approach [10, 11]. An enrichment design provides the prospective use of the patient characteristic to select a study population in which detection of a treatment effect is more likely than it would be in an unselected population [12].

The Food and Drug Administration (FDA) guidelines consider the ML as a suitable method to enhance patient cohort selection (i) by reducing the sample heterogeneity, (ii) by choosing patients who are more likely to have a measurable clinical endpoint (prognostic enrichment), and (iii) by identifying a population more capable of responding to treatment, also termed (predictive enrichment) [10, 11]. In addition, the European Medicines Agency (EMA) also recommends collaborating with the trial stakeholders to design collaborative clinical trials to innovate and expedite patient identification procedures [13].

Despite the international guidelines indications, these methods have been applied rarely. However, some application examples can be found in neurological trials on Alzheimer's disease [14] and cognitive impairment [15].

The literature reports the potentialities of ML in aiding the patient selection process in clinical trials. However, the method finds little application except in the initial stages of patient recruitment [16]. The patients' enrollment in a clinical trial could constitute a complex issue because of the complex inclusion criteria and the additional workload that a systematic patient search could involve for a physician [17]. The pre-screening process is automatized in several settings by using the ML methods; for example, different automated clinical trials eligibility screening tools have been proposed in the literature [18].,

Despite this, using MLT techniques as a method to automate the run-in process, making it less time- and cost-consuming in clinical trials, is little addressed in the literature.

For this reason, we propose an ML predictive capabilities exploitation in clinical trials during early accrual in the spirit of a population enrichment approach.

With this purpose, an ensemble model of ML techniques was trained on a couple of twin randomized clinical trials to learn from one trial data and to mimic a run-in process on the other one. This work proposes an innovative and efficient run-in method in clinical trials that combines the possibility of optimizing the probability that a patient could benefit from the study treatment with a considerably less time- and cost-consuming approach than the traditional run-in.

The ML-enforced run-in issue has been introduced in the “Introduction” section (paragraph 1). The “Materials and Methods” section (paragraph 2) is composed of the case study description (2.1), together with an overview of the dataset variable collected at baseline (2.2) and the trial outcome assessment (2.3). The SL algorithm description is reported in subsection (2.4) with the procedure performed to mimic the SL-enforced run-in phase (2.5) with the algorithm implementation in an R [19] environment in subparagraph (2.6), imputation method (2.7), implementation (2.8), and feature selection procedure (2.9). The “Results” are reported in paragraph 3 by reporting the data description results (3.1) with the SL cross-validation results (3.2) and prediction performance (3.3). The relations between the ML run-in assisted proposal and the available literature has been reported in “Discussion” (paragraph 5), underling the limitation and potentiality in the “Conclusion” section (paragraph 6).

## 2. Materials and Methods

### 2.1. Case Study

A couple of twin clinical trials that (subsequently identified with A and B) consist of two small short-term trials on knee osteoarthritis are considered. At the time of writing, authors are not entitled to disclose all the data information due to confidentiality reasons.

Both trials are randomized, double-blinded, parallel, and placebo-controlled to assess the superiority of the same pharmacological treatment for knee osteoarthritis on symptoms' modification at six months of follow-up, measured on the Western Ontario and McMaster Universities (WOMAC) scale [20].

The treatment and placebo arms were sealed package so they were indistinguishable. Patients were randomized to the intervention groups by using computer-generated random numbers. The treatment encoding assigned to each patient was stored in an opaque sealed envelope and only opened in case of emergency.

### 2.2. Dataset and Variables Collected at Baseline

Overall, the analysis of ML predictive capabilities as a run-in period replacement considered 257 patients (120 from trial A and 137 from trial B) with several baselines demographic, lifestyle, and clinical characteristics, including smoking habit, alcohol consumption, caffeine consumption, diet's type, body mass index (BMI), blood pressure, concomitant infectious diseases, concomitant medications, and signs and symptoms of knee osteoarthritis like knees' erythema, temperature increase, effusion, bony enlargement, and knees' degree of flexion and alignment. The distributions of the baseline characteristics in each trial are shown in Table 1. Further details concerning the patient's characteristics for the twin trial within the treatment arms have been reported in Supplementary Material (Table S1).

To better clarify the relationship between the variables, a synthetic dataset that mimics the structure of the original data frame has been attached as additional material (“sint_db.txt”). The procedure used to create the dataset is presented in the Supplementary 2.

### 2.3. Outcome Assessment

Symptoms' modification during the observation period was assessed by measuring (at baseline and follow-up visits) the WOMAC index by the Visual Analogue Scale (VAS) version of the index in both studies (100 mm VAS for each question; the total score is represented by the sum of all the 24 items scores). It is a tri-dimensional, disease-specific, health status measure, assessing symptoms in the areas of pain (5 questions, score range: 0 mm–100 mm each one), stiffness (2 questions, score range: 0 mm–100 mm each one), and physical function limitation (17 questions, score range: 0 mm–100 mm each one). Thus, a higher WOMAC partial/total score represents worse symptoms/situation, with 2400 mm being the worst possible total score.

The primary study outcome consists of the six months comparison (mean difference) of the delta WOMAC between treatment arms.

The secondary outcomes are the 12 and 24 months comparison of the delta WOMAC across treatment arms. The trial design also assesses the variation in osteoarthritis symptoms between Verum and Placebo's arms on pain stiffness and physical function subscales as secondary endpoints.

For the study, we considered the negative variation of total normalized (i.e., with the total length 0-2400 mm rescaled to 0-100) WOMAC index at six months against the one measured at the baseline. The variation was considered a dichotomous variable, using the adequate cut-off levels concerning the WOMAC index at the baseline [21] to reveal a Minimal Clinically Importance Improvement (MCII), i.e., -2.6 mm cut-off for Low Baseline indexes (less than or equal to 35.3 mm) -14.8 mm cut-off for Intermediate indexes (from greater than 35.3 mm and less than or equal to 51.4 mm) and -15.1 mm cut-off for High indexes (greater than 51.4 mm). This way, a patient with a delta total WOMAC score (at six months) lower or equal to the cut-off level referred to their baseline level is considered a responder; otherwise, they are considered a non-responder.

### 2.4. SuperLearner

SuperLearner (SL) is an ensemble of ML techniques combined so it is theoretically proved as being asymptotically as good as the oracle selector, i.e., the best possible weighted combination of the base learners [22].

To develop a Super Learner algorithm, it is necessary to define a library of learners (Ψ_1_, ⋯, Ψ_*L*_), specifying a meta-learning method *Φ* and get a partition of the training observation into *V*-folds (in the current application *V* =5) to carry out the cross-validation for the performance evaluation. With these notations, SL works as follows: It generates a matrix *Z* of size *n* × *L* of cross-validated predictions, i.e., during the cross-validation, it obtains fits ${\hat{\Psi }}_{-v}^{l}$ defined as fitting ${\overset{\wedge }{\Psi }}^{l}$ that are not in the *V*^th^ fold and generates predictions for the observations in the *V*^th^ fold. Next, it finds the optimal combination of subset-specific fits according to the specified meta-learners algorithm $\hat{\Phi }$ with a new matrix *Z* and finally, it fits *L* models, one for each base learning algorithm, on the original training set *X* and it saves the *L* individual model fit objects along with $\hat{\Phi }$. SL also envisages the use of weights for some algorithms. The ensemble model obtained can be used to make predictions on the new data. For a sample size of about 50-70 patients, it is suggested to use *V* =5 or *V* =10 depending on whether we aim to contain the bias or variance, respectively [23]. To apply SL on a population with slightly different characteristics for the training set, we have chosen *V* =5.

The SL method combines several ML algorithms in a (convex) weighted combination of separate algorithms. The weights are selected to minimize the cross-validation error. Once the optimal combinations of the algorithm have been selected, an increase in the number of learners does not affect the SL performance because uninformative learners are zero weighed [22].

### 2.5. Procedure

Patients enrolled in trial A were used to train two SLs at predicting variation of WOMAC score at six months: one SL was trained on patients enrolled in the placebo arm and the other one on patients in the experimental arm. Then for validation purposes, the algorithms developed on patients in trial A were validated using them to predict the outcome at six months on patients enrolled in trial B.

The same procedure was applied by reversing A and B, i.e., two SLs were trained on patients enrolled in trial B to predict the outcome of patients in trial A.

Sensitivity (Sen), Specificity (Spec), Positive and Negative Predictive Value (PPV and NPV), Accuracy (ACC), the Area Under the Receiver Operating Characteristic (ROC) Curve (AUC), and the ROC were reported to assess the performance of the procedure.

To simulate a run-in period for both arms, we have assumed that the randomization process was well balanced for each trial. This approach was necessary to consider the population in each arm as they represent an independent population. Hence, four SLs were trained: two to predict placebo run-in and two to predict Verum run-in processes.

### 2.6. Base Learner Algorithms

The following provides a short description of the ML algorithms used as base learners. The algorithms are selected to include the most common technique used in non-deep machine learning models across the ones already implemented in the SuperLearner R package.

*Classification and Regression Trees (CART)* [24] are methods to fitting models obtained by recursively partitioning the data and fitting simpler models within each partition. As a result, the partitioning can be represented graphically as a decision tree.

*Random Forest (RF)* [25] recursively creates multiple decision trees. The training process selects a subset of available features and recursively partitions the data until the subspace variation is slight. As a greedy technique, RF does not necessarily converge to an optimal global solution. For avoiding such an indecisive convergence, a collection or ensemble of locally optimal trees can be done (bagging.) The ensemble of those trees is “the forest.”

*Bagging Trees* is an ML that falls into the category of ensemble learning. Several CART algorithms are trained on different datasets in bagging, each one obtained from the initial dataset through random sampling with replacement (bootstrap). The name bagging derives from the combination of the words bootstrap (that is, the random sampling with replacement) and aggregation (referring to the aggregation of more models, typical of ensemble learning) [26].

*Gradient Boosting Machines (GBM)* [27] construct tree-based models on the residuals using the specified list of variables. Next, they explain the variance in the residuals. The total number of trees specified for the model building was 500 with an interaction depth of five, and the learning weight of iteration was 0.1.

*Generalized Linear Model (GLM) with elastic net regularization* [28] is a regularized regression algorithm that linearly combines the L1 (lasso) and L2 (ridge) penalties in synergy with a link function to overcome the linear model limitation.

*Polychotomous regression or classification based on Multivariate Adaptive Regression Splines (POLYMARS)* [29] uses linear splines and selected tensor products to fit multiple classifications to avoid estimating pure multiple classification methods focusing on the estimation of reliable conditional class probabilities for the classification.

### 2.7. Missing Data

At the time of writing, SL cannot handle missing data. Enforcing a Multivariate Imputations by Chained Equations (MICE) approach [30], we have performed five multiple imputations with a monotone visit sequence, i.e., the variables are sorted by the increasing amount of “missingness” to impute the data during each step (of the five) through the data. The function used to perform the imputation is provided by the *mice* R package [31].

### 2.8. Implementation

SuperLearner [32] R [19] package is available at the CRAN (The Comprehensive R Archive Network), and the functions implemented within it are the ones that were used to train the SL MLTs. SuperLearner requires the specification of all the candidate algorithms which constitute the ensemble model. The algorithms used by us and combined in the SuperLearner are the ones that can manage categorical data that are included in the “SL.complete. library” library provided by the package. The final set of algorithms is formed by:

- The “SL.caret. rf” and “SL.caret.rpart” are the SuperLearner functions implementing the RF and CART (or RPART, recursive partitioning for classification and regression trees) algorithms, respectively, by considering the caret [33] package environment. The “SL.rpartPrune” algorithm has also been considered. The function uses nested sequences of subtrees by recursively snipping off the least important splits regarding their complexity. The “SL.randomForest” and “SL.rpart,” respectively, implementing the RF and CART algorithm in the Random Forest [34] and rpart [35] packages have also been considered
- The bagging tree algorithm [26, 36] has been implemented with “SL.ipredbagg” interfacing with ipred [37] R package
- “SL.polymars” is the function implementing POLYMARS algorithm by using the MARS [38] (Multivariate Adaptive Regression Splines) algorithm with the function “SL.earth”
- “SL.gbm” implements the GBM function
- “SL.glmnet” function implements GLM with elastic net regularization
- “SL.mean” is the simple weighted mean of the outcome predictions

### 2.9. Feature Selection

All the algorithms were computed based on the set of variables and on the subsets selected by the screening algorithm “screen. randomForest,” which uses the Random Forest algorithm for the variable selection. Overall, 22 (i.e., 11 x 2) different algorithms were evaluated to be ensemble all together into each of the four SuperLearner trained.

The algorithms have been trained both on the overall set of predictors and on a subset of relevant features for the ensemble SL and separate learners. The features have been selected by considering the mean decrease in accuracy of a RF algorithm. The performance for the considered models is reported in Table 2 by identifying the model with the rule “SL, {Name of algorithm},{selected predictors}.” For example, the label “SL, Mars Algorithm, all features” indicates the performance (defined as the average value of MSE in the Cross-Validation procedure) of a single Mars algorithm trained by including as predictors all the candidate features. The “SL, Mars Algorithm, RF screened features” label instead indicates the Mars algorithm performance computed by including a subset of relevant features selected by the RF Variable importance measure. Moreover, the notation “SL, average, all features” indicates the performance of the overall ensemble SL (average) trained by considering all the candidate predictors.

## 3. Results

### 3.1. Data Description

The analyses have been performed on 257 patients (120 from trial A and 137 from trial B). The median age is 63 years in both arms for trial A and 65 for trial B in all the treatment groups. The study sample is mainly composed of females. The median Body Mass Index indicates overweight stratus (BMI>25) in all the treatment groups for both studies.

The percentage of therapy responder is 50% in the placebo group and 62% in the Verum arm for study A. The response rate of trial B is 49% in the placebo arm and 55% in the Verum group (Table 1). Other study characteristics ate reported in the Supplementary Material (Table S1).

### 3.2. SuperLearner

Table 2 shows the retained learners that constitute the final ensemble algorithm for each of the four prediction tasks.

Moreover, for each of the formed SLs, Table 2 reports the risk associated with each base learner (i.e., an average of the mean squared errors among the cross-validated algorithms, the lower, the better) and the weight of each learner within the given SL. Weights equal to zero are omitted. Notably, every task differs from the others in the chosen technique.

### 3.3. Prediction

Table 3 reports the testing performance statistics for outcome predictions on each arm of one study for each SLs trained on the corresponding arm of the other study.

**Figure 1:**
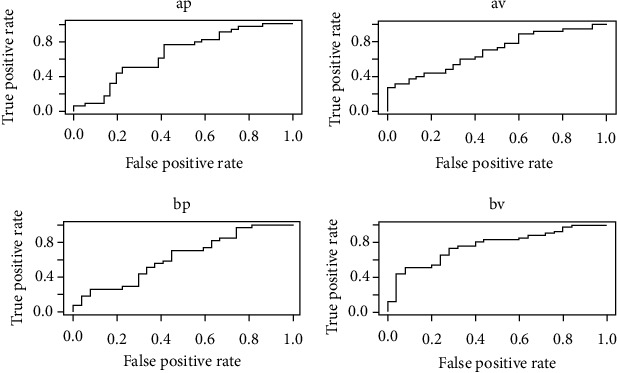
ROC curves for the SL performance. ROC curves for the SL performance. ap = SL trained on trial A placebo, tested on trial B placebo; av = SL trained on trial A Verum, tested on trial B Verum; bp = SL trained on trial B placebo, tested on trial A placebo; bv = SL trained on trial B Verum, tested on trial A.

Corresponding resulting ROC (Receiver Operating Characteristic) curves are reported in Figure 1.

The results obtained are above 70% by correctly detecting Verum responders when the SL is trained on the first trial and tested on the second and vice-versa and are above 80% to be in the right when marking a patient on the Verum arm as non-responders using the second trial SL and of 69% using the first one for the train. On the other side, the probability of detecting non-responder correctly for the placebo arm is over 70% only in one direction, i.e., when the SL is trained on the second trial and tested on the first. While on the reverse direction, the performance remains similar to a coin tossing. For the PPV for the placebo arms, the performance is near 60%. By looking at the sample sizes, and especially regarding the statistics of interest mentioned, it emerges that the SL trained on trial.

## 4. Discussion

This study illustrates the application of an SL algorithm for the early prediction of patients' outcomes, which could be helpful as a potential replacement for the run-in period. The management and conduction costs are one of the possible issues to be faced when conducting a clinical trial. The run-in procedure involves additional costs and time to the study conduction [39]. The use of MLT techniques could automate the run-in selection process.

The literature demonstrated that the improvement of the human abilities with ML could involve significant enhancement, over the classical procedure, of the patient pre-screening process, substantially increasing the number of patients eligible for the trial enrollment. Moreover, the procedure reduces the person-hours required and the elapsed time between patient eligibility assessment and the final enrollment [40].

In contrast, the proposed use of MLT techniques to mimic the run-in process remains poorly investigated in the literature. However, our results highlight an approach that may prove promising for clinical trial design.

The prediction based on known information about the same given therapy (Placebo or Verum) on the same amount of time in a similar population concerning eligible criteria can assess a good approximation of a real run-in, e.g., a trained SL on patients treated with the placebo is supposed to have learned “the way the placebo influences the population's outcome” in such a period considering the predictors provided.

In the case study, we have modeled the working pattern of the placebo arm and the Verum one, testing the model on a similar population, achieving a moderate discrimination ability (i.e., AUC from 63% to 76%).

However, the distribution of the baseline characteristics highlights balanced randomization of the patients in each trial but not that good similarity between the “twins.” This result enforces our assumption that it is possible to consider only the patients in one arm as a distinct population in this situation.

Each study was divided into two parts for what concerns the arm, and each used to train a classifier. So, the classes' sizes were reduced to a minimum of 54 patients for the placebo arm (study A) and a maximum of 70 patients for the placebo arm (study B). Regarding variance (high when the cases in each fold of the cross-validated training sets are too much different from each other) and bias (high when the cases in each training set are too similar to each other) trade-off, relying on those figures makes cross-validation difficult concerning the number of folds to specify [41].

On one side, this could be seen as a contextual issue, and on the other side, it shows how this procedure, particularly the SL, performs well even with pretty small sample size. Regardless, data sharing will allow researchers to overcome this limitation from a collaborative perspective and achieve better results.

### 4.1. Potential of SL for Use in Run-In Selection

A fine-tuned SL could be successfully exploited to forecast the possible run-in results of a clinical trial, provided that (i) its prediction capability has been assessed on an independent test data and it reaches the desired levels; (ii) appropriate data is available, e.g., containing the predictor and outcome variables required for the prediction model; and (iii) the use of such predictions will not introduce any selection bias in the clinical trial process. For example, Figure S1 (Supplementary Material) depicts a hypothetical example of a run-in period envisaged to screen out patients who fail to respond at a short-term evaluation, thus are not suitable for the long-term evaluation, which is the trial's primary objective. In this case, the application of SL before randomization (Figure S2, Supplementary Material) could help excluding patients failing in the short term by tightening cost and time without introducing a bias.

The strength of the procedure is in the long-term application and team/consortium work: an SL, trained on a first trial and validated on a second similar one, can be used to predict the outcome of other trials. This way, every member of the consortium could use the trained SL to anticipate the outcome of its subsequent trial's run-in process, thus excluding patients with a high probability to be non-responders (NPV) under treatment and to have the dually high probability that a true responder will be marked as responder (Sens). On the other side, it is advisable to have a high probability of correctly detecting a non-responder (Spec) under the placebo and ensuring that a patient marked as a responder under placebo is likely to be true (PPV).

In this context, automatic implementation of run-in would greatly speed up enrollment procedures. Moreover, a further potential of the method is that the MLT system could learn to predict the patient's responsiveness to therapy not only from the information available within the trial being conducted but also using data from trials conducted in similar experimental settings. All of this could be useful in building increasingly efficient and accurate predictive machines.

The run-in process is used in clinical trials to exclude patients after the selection procedure before the randomization. The procedure could improve the probability of detecting a treatment effect [42]. In this regard, the literature demonstrated that ML algorithms could assist the trial conduction by supporting the patient recruitment process. The ML algorithms could enhance the trial selection as addressed by the Food and Drug Administration (FDA) [43] by limiting the heterogeneity of the sample selecting the patients who are more likely to result in an observable outcome (prognostic enrichment). Moreover, it is recognized also the ML role in the predictive enrichment process by improving the possibility of identifying a sample of patients more capable of responding to the treatment [11].

There is a considerable variety of ML reported in the literature according to their different properties and characteristics; the SL ensemble allows combining many candidate algorithms. Some authors demonstrate that the SL generally performs better in comparison with the separate ML learners. The SL ensemble also has an important practical advantage limiting the need for trial planners to choose among different ML algorithms, because all of them could be considered for developing an SL [44].

The regulatory agencies have extensively commented on the procedures for conducting classical clinical trial run-in, emphasizing advantages and disadvantages and also suggesting data analysis procedures peculiar to this context [10]. Certainly, the application of MLT techniques in the run-in phase poses new issues to be argued by regulatory agencies as the procedures of MLT algorithms tuning will have to be well established and validated for the real cases of application.

### 4.2. Study Limitation

The predictive performance of the proposed tool is not optimal on the case study considered due to the limited sample size (the computation has been reported in the Supplementary material).

Moreover, the run-in approach could involve a bias in favor of the active treatment [45]. However, the procedure is widely applied in clinical trials to exclude patients who probably would be poor responders or poorly compliant with the therapy. The patient exclusions are used to achieve an enriched study sample with increased treatment response and increased statistical power [46]. Machine learning techniques in the run-in phase, compared to conventional procedures, could improve the patient selection procedure by using several types of information such as textual data, imaging, and device data [11], abating the costs and the times related to run-in. Appropriate selection bias management techniques, for example, based on missing data imputation, are suggested to analyze the run-in trial data accounting for all participants who were intended to be randomized [45].

The quantification of the MLT-based run-in effect on a possible selection bias, in comparison with the traditional procedures, remains an interesting point to be addressed from a clinical and regulatory point of view; few studies report a systematic comparison between the traditional medical-assisted run-in phase and the ML-assisted patient selection procedure. Future research developments are needed to further investigate these aspects according to the clinical trial settings, patients' characteristics, and disease profile.

## 5. Conclusions

The potentiality of the SL-enforced run-in approach consists in the fact that trials conducted in similar settings can be used to train an optimal patient selection algorithm tailored to optimize the treatment response according to the patient's characteristics. In this manner, the developed SL algorithm would mimic a run-in process in a new starting clinical trial.

Moreover, the SL algorithm shows a high grade of adaptability about the possibility to choose the most suitable base learners in each situation, maintaining the possibility to incorporate any newly developed MLT into its library.

## Figures and Tables

**Table 1:** Baseline characteristics, stratified by trial (A or B) and treatment (Placebo or Verum). Continuous variables are expressed in terms of I., II. (median), and III. quartiles while categorical ones with frequencies and absolute values.

Variables	Valid cases	Trial A		Trial B	
		Placebo (*N* =54)	Verum (*N* =66)	Placebo (*N* =70)	Verum (*N* =67)
Age (years)	253	59/63/69	57/63/66	60/65/69	59/65/71
Body mass index	257	26/27/27	24/26/27	26/28/30	25/28/30
Gender: male	257	30%(16)	20%(13)	23%(16)	21%(14)
Height (cm)	257	160/165/173	163/166/170	154/160/165	154/160/166
Weight (kg)	257	66/71/79	65/70/75	64/69/77	63/70/77
Therapy responder	257	50%(27)	62%(41)	49%(34)	55%(37)

**Table 2:** Base learner used for each SL trained; risk (average value of MSE in the Cross-Validation procedure) and coefficient (weight of the base learner convex combination used to form the SL) are reported. Weights equal to zero are omitted. The algorithm composing the SL is identified; the average indicates the SL average ensemble prediction algorithm. The screening (feature selection) algorithm has been also identified. For example, “*SL, Mars Algorithm, RF screened features”* identify the risk associated with the Mars algorithm within SL ensemble with an RF-based feature selection procedure.

SL trained on study A – Placebo	Risk	Coefficient
SL, Mars Algorithm, all features	0.177	0.213
SL, Mars Algorithm, RF screened features	0.161	0.257
SL, average, all features	0.139	0.311
SL, Rpart, RF screened features	0.150	0.219

SL trained on study A – Verum	Risk	Coefficient
SL, average, all features	0.121	0.539
SL, Polymars, RF screened features	0.131	0.410
SL, RF, RF screened features	0.132	0.051

SL trained on study B – Placebo	Risk	Coefficient
SL, Mars Algorithm, all features	0.099	0.170
SL, Glmnet Algorithm, all features	0.082	0.119
SL, Glmnet Algorithm, RF screened features	0.075	0.298
SL, average, all features	0.127	0.015
SL, RF, RF screened features	0.076	0.398

SL trained on study B - Verum	Risk	Coefficient
SL, Rpart, all features	0.126	0.124
SL, average, all features	0.127	0.523
SL, Polymars, RF screened features	0.191	0.141
SL, RF, RF all features	0.126	0.213

Abbreviations: SL = SuperLearner; RF = Random Forest; Glmnet = Lasso and Elastic-Net Regularized 329 Generalized Linear Models; Mars = Multivariate Adaptive Regression Splines; Polymars = Poly-330 chotomous classification based on Multivariate Adaptive Regression Splines; Rpart = Recursive Par-331 titioning Trees.

**Table 3:** Predictive performance statistics. The sentence “X to Y” (where X is trial A or trial B and Y is the other trial) indicates the performance of an algorithm trained on study X and tested on study Y (only on the indicated arm).

	Sens	Spec	Acc	PPV	NPV	AUC
A to B Placebo	0.611	0.529	0.571	0.579	0.563	0.658
B to A Placebo	0.370	0.778	0.574	0.625	0.553	0.630
A to B Verum	0.700	0.541	0.612	0.553	0.690	0.693
B to A Verum	0.760	0.634	0.682	0.559	0.813	0.763

Sens = sensitivity; Spec = specificity; Acc = accuracy; PPV=Positive Predictive Values; NPV=Negative Predictive Values; AUC = Area Under Curve.

## Data Availability

Data available upon reasonable request to the authors.

## Supplementary Materials

Supplementary 1 Table S1 Baseline characteristics, stratified by trial (A or B) and treatment (Placebo or Verum). Continuous variables are expressed in terms of I., II. (median), and III. quartiles while categorical ones with frequencies. LK = Left Knee; RK = Right Knee; OA = Osteoarthritis; Y = Yes. Supplementary material Figure S1: Classical paradigm implementation of a run-in period into a clinical trial workflow. Supplementary material Figure S2: SuperLearner paradigm proposal to substitute a run-in period for all the trial, in a framework of study with similar population and treatment, out of the one(s) selected to train the SuperLearner itself. Supplementary material Figure S3 Comparison of variable distributions among the synthetic and observed data.

Supplementary 2 Synthetic dataset in txt format.

## References

[1] Rathod T., Belcher J., Montgomery A. A., Salisbury C., Foster N. E. (2014). Health services changes: is a run-in period necessary before evaluation in randomised clinical trials?. *Trials*.

[2] Pablos-Méndez A., Barr R. G., Shea S. (1998). Run-in periods in randomized trials. *JAMA*.

[3] Berger V. W., Durkalski V. L. (2014). *Run-In Period*.

[4] Fitzpatrick A. M., Jackson D. J., Mauger D. T. (2016). Individualized therapy for persistent asthma in young children. *The Journal of Allergy and Clinical Immunology*.

[5] Witten I. H., Frank E. (2005). *Data Mining: Practical Machine Learning Tools and Techniques*.

[6] Flach P. (2012). *Machine Learning: The Art and Science of Algorithms That Make Sense of Data*.

[7] Oermann E. K., Rubinsteyn A., Ding D. (2016). Using a machine learning approach to predict outcomes after radiosurgery for cerebral arteriovenous malformations. *Scientific Reports*.

[8] Kourou K., Exarchos T. P., Exarchos K. P., Karamouzis M. V., Fotiadis D. I. (2015). Machine learning applications in cancer prognosis and prediction. *Computational and Structural Biotechnology Journal*.

[9] Shouval R., Bondi O., Mishan H., Shimoni A., Unger R., Nagler A. (2014). Application of machine learning algorithms for clinical predictive modeling: a data-mining approach in SCT. *Bone Marrow Transplantation*.

[10] US Food and Drug Administration (2019). *Enrichment Strategies for Clinical Trials to Support Determination of Effectiveness of Human Drugs and Biological Products Guidance for Industry*.

[11] Harrer S., Shah P., Antony B., Hu J. (2019). Artificial intelligence for clinical trial design. *Trends in Pharmacological Sciences*.

[12] Simon N., Simon R. (2013). Adaptive enrichment designs for clinical trials. *Biostatistics*.

[13] Hines P. A., Gonzalez-Quevedo R., Lambert A. I. (2020). Regulatory science to 2025: an analysis of stakeholder responses to the European Medicines Agency’s strategy. *Frontiers in Medicine*.

[14] Ithapu V. K., Singh V., Johnson S. C. (2017). Randomized deep learning methods for clinical trial enrichment and design in Alzheimer’s disease. *Deep Learning for Medical Image Analysis*.

[15] Ithapu V. K., Singh V., Okonkwo O. C. (2015). Imaging-based enrichment criteria using deep learning algorithms for efficient clinical trials in mild cognitive impairment. *Alzheimer’s & Dementia*.

[16] Liu R., Rizzo S., Whipple S. (2021). Evaluating eligibility criteria of oncology trials using real-world data and AI. *Nature*.

[17] Cuggia M., Campillo-Gimenez B., Bouzille G. (2015). Automatic selection of clinical trials based on a semantic web approach. *Studies in Health Technology and Informatics*.

[18] Penberthy L., Brown R., Puma F., Dahman B. (2010). Automated matching software for clinical trials eligibility: measuring efficiency and flexibility. *Contemporary Clinical Trials*.

[19] R Core Team R (2015). *A Language and Environment for Statistical Computing; R Foundation for Statistical Computing*.

[20] Bellamy N. (2002). WOMAC: a 20-year experiential review of a patient-centered self-reported health status questionnaire. *The Journal of Rheumatology*.

[21] Tubach F., Ravaud P., Baron G. (2005). Evaluation of clinically relevant changes in patient reported outcomes in knee and hip osteoarthritis: the minimal clinically important improvement. *Annals of the Rheumatic Diseases*.

[22] van der Laan M. J., Polley E. C., Hubbard A. E. S. (2007). Super Learner. *Statistical Applications in Genetics and Molecular Biology*.

[23] Friedman J., Hastie T., Tibshirani R. (2001). *The Elements of Statistical Learning*.

[24] Breiman L., Friedman J. H., Olshen R. A., Stone C. J. (1984). *Classification and Regression Trees*.

[25] Breiman L. (2001). Random Forests. *Machine Learning*.

[26] Breiman L. (1996). Bagging predictors. *Machine Learning*.

[27] Friedman J. H. (2001). Greedy function approximation: a gradient boosting machine. *The Annals of Statistics*.

[28] Friedman J., Hastie T., Tibshirani R. (2010). Regularization paths for generalized linear models via coordinate descent. *Journal of Statistical Software*.

[29] Kooperberg C., Bose S., Stone C. J. (1997). Polychotomous regression. *Journal of the American Statistical Association*.

[30] Buuren S. (2012). *Flexible imputation of missing data*.

[31] Van Buuren S., Groothuis-Oudshoorn K., Robitzsch A. (2011). Mice: multivariate imputation by chained equations inR. *Journal of Statistical Software*.

[32] Polley E., LeDell E., Kennedy C., Lendle S., van der Laan M. (2019). *Package ‘SuperLearner’*.

[33] Kuhn M. (2008). Building predictive models in R using the caret package. *Journal of Statistical Software*.

[34] Liaw A., Wiener M. (2002). Classification and regression by RandomForest. *R news*.

[35] Therneau T., Atkinson B. (2019). *Rpart: recursive partitioning and regression trees*.

[36] Breiman L. (1996). *Out-of-bag estimation*.

[37] Peters A., Hothorn T. (2019). *ipred: Improved Predictors [Computer Software Manual]*.

[38] Milborrow S. (2021). Derived from mda:mars by Trevor Hastie and Rob Tibshirani, Uses Alan Miller’s Fortran utilities with ThomasLumley’s leaps wrapper. *Earth: Multivariate Adaptive Regression Splines*.

[39] Arenz D., Hero B., Eichhorst B. F. (2014). Estimating site costs prior to conducting clinical trials. *Clinical Investigation*.

[40] Calaprice-Whitty D., Galil K., Salloum W., Zariv A., Jimenez B. (2020). Improving clinical trial participant prescreening with artificial intelligence (AI): a comparison of the results of AI-assisted vs standard methods in 3 oncology trials. *Therapeutic Innovation & Regulatory Science*.

[41] Molinaro A. M., Simon R., Pfeiffer R. M. (2005). Prediction error estimation: a comparison of resampling methods. *Bioinformatics*.

[42] Laursen D. R. T., Paludan-Müller A. S., Hróbjartsson A. (2019). Randomized clinical trials with run-in periods: frequency, characteristics and reporting. *Characteristics and Reporting. CLEP*.

[43] Vamathevan J., Clark D., Czodrowski P. (2019). Applications of machine learning in drug discovery and development. *Nature Reviews. Drug Discovery*.

[44] Zhang Z., Ma S. (2019). Machine learning methods for leveraging baseline covariate information to improve the efficiency of clinical trials. *Statistics in Medicine*.

[45] Berger V. W., Vali B. (2011). Intent-to-randomize corrections for missing data resulting from run-in selection bias in clinical trials for chronic conditions. *Journal of Biopharmaceutical Statistics*.

[46] Schechtman K. B. (2017). Run-in periods in randomized clinical trials. *Journal of Cardiac Failure*.

