# Supplementary material for: A SuperLearner Approach to Predict Run-In Selection in Clinical Trials
Source: Comput Math Methods Med. 2022 Sep 10;2022:4306413. doi: 10.1155/2022/4306413 (PMC9482682; doi:10.1155/2022/4306413)
Supplement: Supplementary 1 — Table S1 Baseline characteristics, stratified by trial (A or B) and treatment (Placebo or Verum). Continuous variables are expressed in terms of I., II. (median), and III. quartiles while categorical ones with frequencies. LK = Left Knee; RK = Right Knee; OA = Osteoarthritis; Y = Yes. Supplementary material Figure S1: Classical paradigm implementation of a run-in period into a clinical trial workflow. Supplementary material Figure S2: SuperLearner paradigm proposal to substitute a run-in period for all the trial, in a framework of study with similar population and treatment, out of the one(s) selected to train the SuperLearner itself. Supplementary material Figure S3 Comparison of variable distributions among the synthetic and observed data. [file 4306413.f1.docx]

**Supplementary Material**

Table S1 Baseline characteristics, stratified by Trial (A or B) and Treatment (Placebo or Verum). Continuous variables are expressed in terms of I., II. (median), and III. quartiles while categorical ones with frequencies. LK = Left Knee; RK = Right Knee; OA = Osteoarthritis; Y = Yes.

|  | **N** | **A: PLACEBO** | **A: VERUM** | **B: PLACEBO** | **B: VERUM** |
| --- | --- | --- | --- | --- | --- |
|  |  | **(N=54)** | **(N=66)** | **(N=70)** | **(N=67)** |
| **Alcohol:Y** | 257 | 37%(20) | 36%(24) | 53%(37) | 57%(38) |
| **Caffeine:Y** | 257 | 81%(44) | 80%(53) | 91%(64) | 90%(60) |
| **Smoking:Y** | 257 | 17%(9) | 11%(7) | 17%(12) | 22%(15) |
| **Systolic Blood Pressure** | 257 | 130.00/136.50/145.00 | 120.00/135.00/148.75 | 125.00/135.00/150.00 | 130.00/140.00/157.50 |
| **Pulse Rate** | 257 | 66.0/72.0/80.0 | 64.5/70.0/76.0 | 60.0/68.0/72.0 | 65.5/72.0/80.0 |
| **Diastolic Blood Pressure** | 257 | 75.00/85.00/90.00 | 71.25/80.00/90.00 | 75.00/80.00/90.00 | 80.00/85.00/90.00 |
| **Pooled Diet: NORMAL** | 257 | 91%(49) | 88%(58) | 84%(59) | 78%(52) |
| **Drug allergies:Y** | 257 | 22%(12) | 21%(14) | 17%(12) | 18%(12) |
| **Non drug allergies:Y** | 257 | 20%(11) | 14%(9) | 26%(18) | 18%(12) |
| **Serious infection:Y** | 256 | 11%(6) | 8%(5) | 39%(27) | 21%(14) |
| **Knee involvment : both** | 257 | 81%(44) | 64%(42) | 94%(66) | 88%(59) |
| **none** |  | 2%(1) | 2%(1) | 0%(0) | 3%(2) |
| **One** |  | 17%(9) | 35%(23) | 6%(4) | 9%(6) |
| **Concomitant medication:Y** | 257 | 72%(39) | 71%(47) | 49%(34) | 49%(33) |
| **Year of knee OA presence** | 257 | 7.00/10.00/14.75 | 5.25/8.50/13.50 | 2.00/5.00/10.00 | 2.50/6.00/10.00 |
| **Palpable effusion (LK):Y** | 253 | 19%(10) | 20%(13) | 33%(23) | 28%(19) |
| **Palpable effusion (RK):Y** | 254 | 32%(17) | 37%(24) | 30%(21) | 30%(20) |
| **Palpable increase in temperature (LK):Y** | 256 | 20%(11) | 28%(18) | 3%(2) | 3%(2) |
| **Palpable increase in temperature (RK):Y** | 257 | 41%(22) | 39%(26) | 3%(2) | 1%(1) |
| **Alignment (LK):NORMAL** | 255 | 59%(32) | 77%(50) | 74%(51) | 78%(52) |
| **VALGUS** |  | 13%(7) | 12%(8) | 23%(16) | 22%(15) |
| **VARUS** |  | 28%(15) | 11%(7) | 3%(2) | 0%(0) |
| **Alignment (RK):NORMAL** | 256 | 63%(34) | 71%(47) | 75%(52) | 81%(54) |
| **VALGUS** |  | 11%(6) | 12%(8) | 19%(13) | 18%(12) |
| **VARUS** |  | 26%(14) | 17%(11) | 6%(4) | 1%(1) |
| **Crepitus active (LK):Y** | 256 | 81%(44) | 88%(57) | 73%(51) | 73%(49) |
| **Crepitus active (RK):Y** | 257 | 78%(42) | 92%(61) | 76%(53) | 76%(51) |
| **Crepitus passive (LK):Y** | 256 | 76%(41) | 75%(49) | 70%(49) | 63%(42) |
| **Crepitus passive (RK):Y** | 257 | 69%(37) | 79%(52) | 71%(50) | 69%(46) |
| **Degree of flexion (LK)** | 256 | 130.00/140.00/140.00 | 135.00/140.00/145.00 | 60.00/70.00/77.75 | 61.50/69.00/74.50 |
| **Degree of flexion (RK)** | 257 | 130.00/140.00/140.00 | 130.00/140.00/143.75 | 60.50/68.50/80.00 | 61.00/70.00/75.00 |
| **Outcomes** |  |  |  |  |  |
| **WOMAC Knee Pain**  **Normalized (0-100)** | 257 | 20.00/30.00/40.00 | 20.00/30.00/40.00 | 18.05/30.40/45.05 | 25.40/38.20/47.80 |
| **(Enrollment Visit)** |  |  |  |  |  |
| **WOMAC Physical Function Normalized (0-100)** | 257 | 16.54/28.68/41.91 | 18.01/30.15/41.91 | 17.66/34.00/56.22 | 25.09/47.18/59.03 |
| **(Enrollment Visit)** |  |  |  |  |  |
| **WOMAC Knee Stiffness Normalized (0-100)** | 257 | 12.50/25.00/37.50 | 15.63/25.00/37.50 | 23.00/49.25/69.63 | 22.50/49.50/72.00 |
| **(Enrollment Visit)** |  |  |  |  |  |
| **WOMAC total Normalized (0-100)** | 257 | 18.75/27.60/39.32 | 19.79/30.21/41.41 | 19.20/33.81/51.95 | 24.56/44.25/56.85 |
| **(Enrollment Visit)** |  |  |  |  |  |


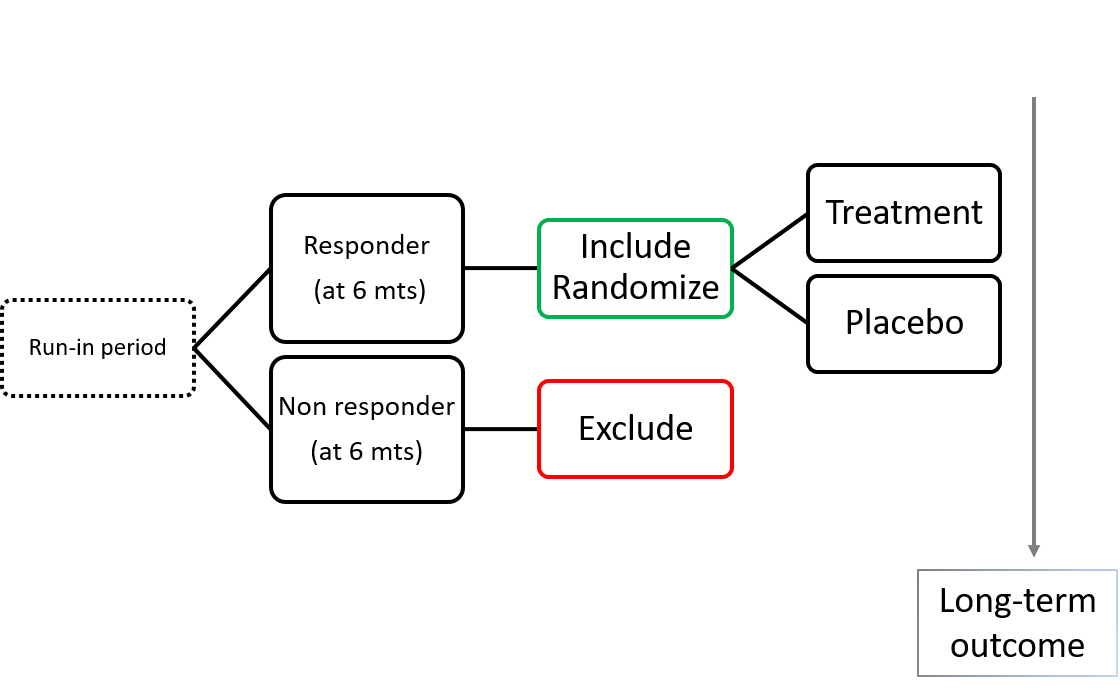


Figure S1: Classical paradigm implementation of a run-in period into a clinical trial workflow.


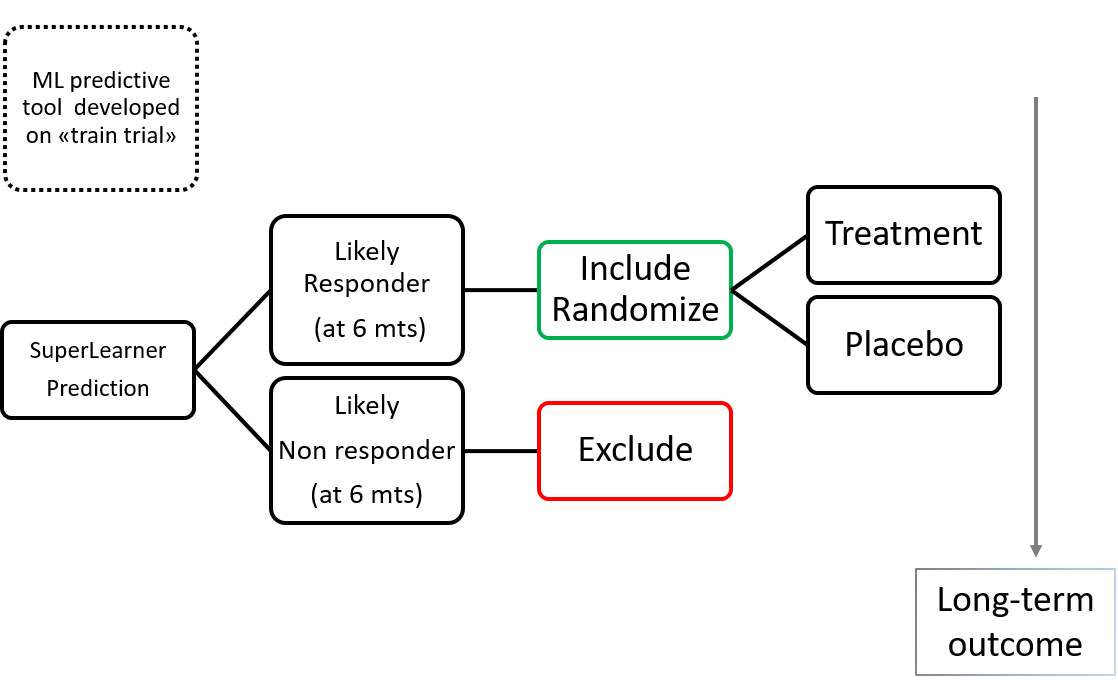


Figure S2: SuperLearner paradigm proposal to substitute a run-in period for all the trial, in a framework of study with similar population and treatment, out of the one(s) selected to train the SuperLearner itself

**General Overview of the SL algorithm**

The basic steps for obtaining predictive estimates of an SL can be summarized as follows ^1^:

1. The procedure starts breaking up the study sample into *V* separate folds
2. For each of the V folds, 1/V samples are considered a validation one and the remaining training ones.
3. Each of the *p* learners composing the SL algorithm is fitted on the training sample.
4. For each observation in the validation sample, the outcome has been predicted by using the single *p* learner. For each patient composing the validation sample, *p* predictions are obtained
5. Another *1/V* fraction of the sample has been considered and repeat until each of the *V*-sets of data are removed.
6. The cross-validated fit of the p learners has been compared across all observations based on specified error function (i.e., the squared-error loss) by calculating the corresponding average loss.

The SL objective is to estimate the function $\psi_{0}(W)=E(Y\mid W),$ which defines the outcome $Y$ expectation conditioned on a set of covariates $W$.

This function is a minimizer of an expected loss $L$ function $\psi_{0}(W)=\mathrm{argmin}_{\psi}E[L(X,\psi(W))]$ which is generally expressed in a quadratic form $L=(Y-\psi(W))^{2}$

1. A weighted average of the SL algorithms is performed to minimize the cross-validated error by re-estimating the algorithms on the original dataset and using the weights mentioned above to obtain the SL fit finally

**Sample size estimation**

The trial data have been simulated by considering the data generation process of a logit model. Five covariates have been included in the simulation strategy by assuming a linear predictor in the form of:

$X\beta=log(Odds) + 3.5*X1 + 0.2*X2 + 2*X3 +5*X4+ 2*X5$.

The compliance probability has been computed as $p= 1/(1 + exp(-X\beta))$, and the simulated trial data have been drawn from a binomial experiment$Y\sim Bin(n,p)$. The baseline Odds=1.17 have been calculated by assuming a 54% compliance rate.

Data have been simulated 100 times; for each simulation run, the SL algorithm has been calculated, and the AUC performance has been stored.

A sample size of $n$ = 250 subjects leads to achieving an average AUC of 0.65 with a bootstrap confidence interval length of 0.11

**Syntetic Dataset**

A synthetic subset of 238 rows has been reported in a .txt file (“sint_db.txt”).

In this research context confidentiality constraints restrict access to unique and

valuable microdata. Synthetic data which mimic the original observed data and preserve

the relationships between variables have been reported. The synthpop^2^ package in R has been considered for the simulation of the data. The CART method (Classification and regression tree) has been used for the data structure development. The comparative plots for the observed and synthetic datasets have been reported below

| 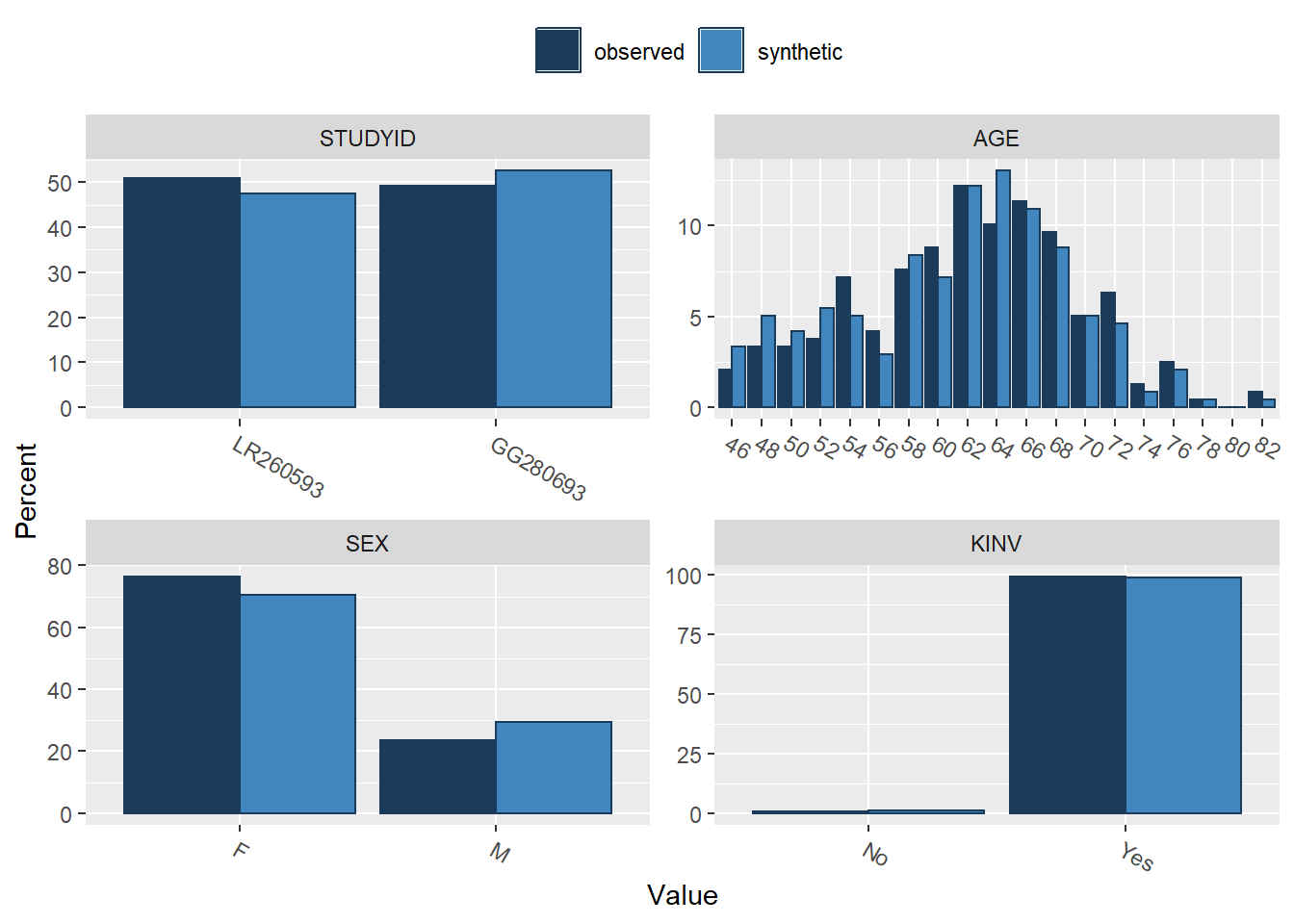 |
| --- |
| 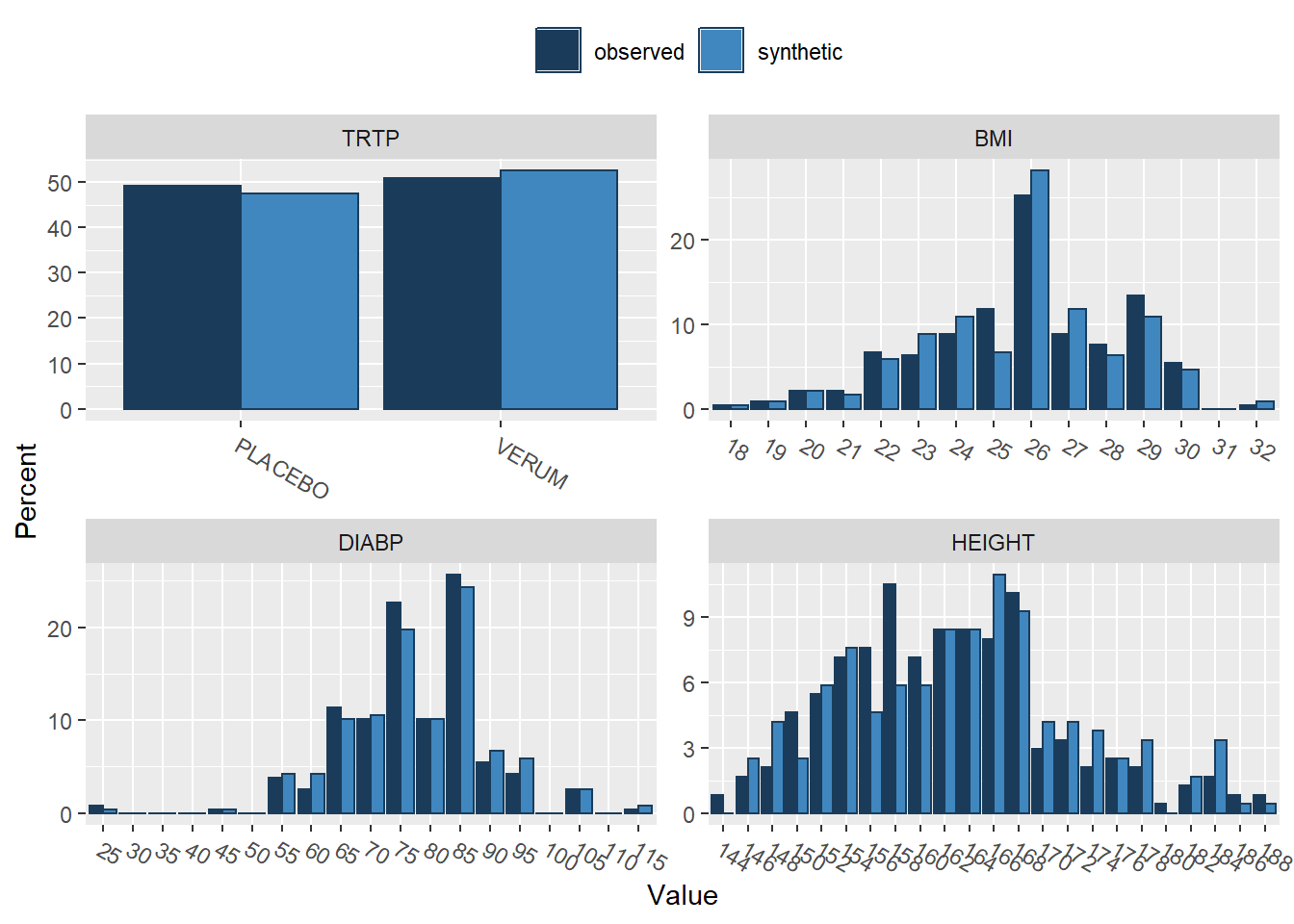 |
| 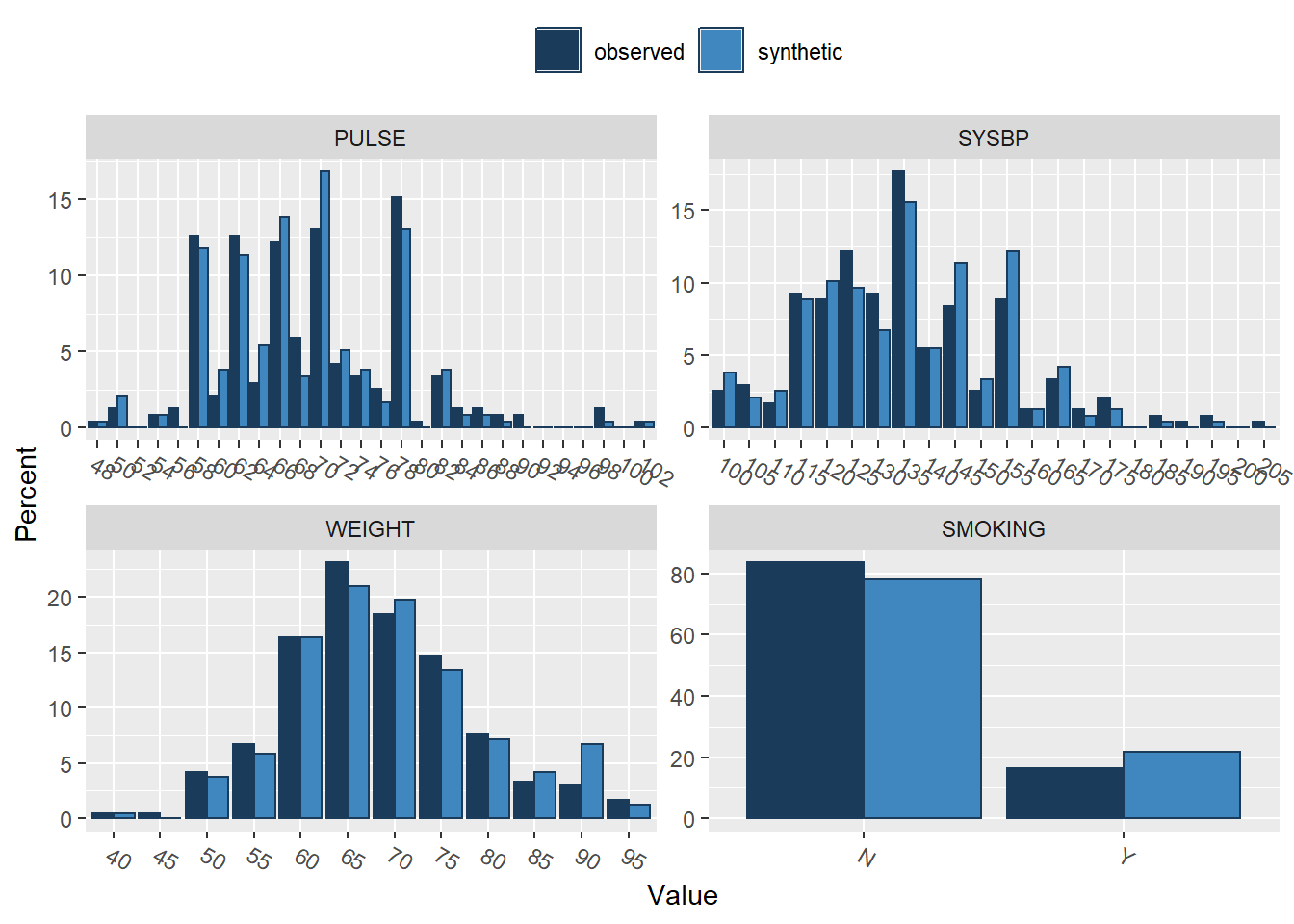 |
| 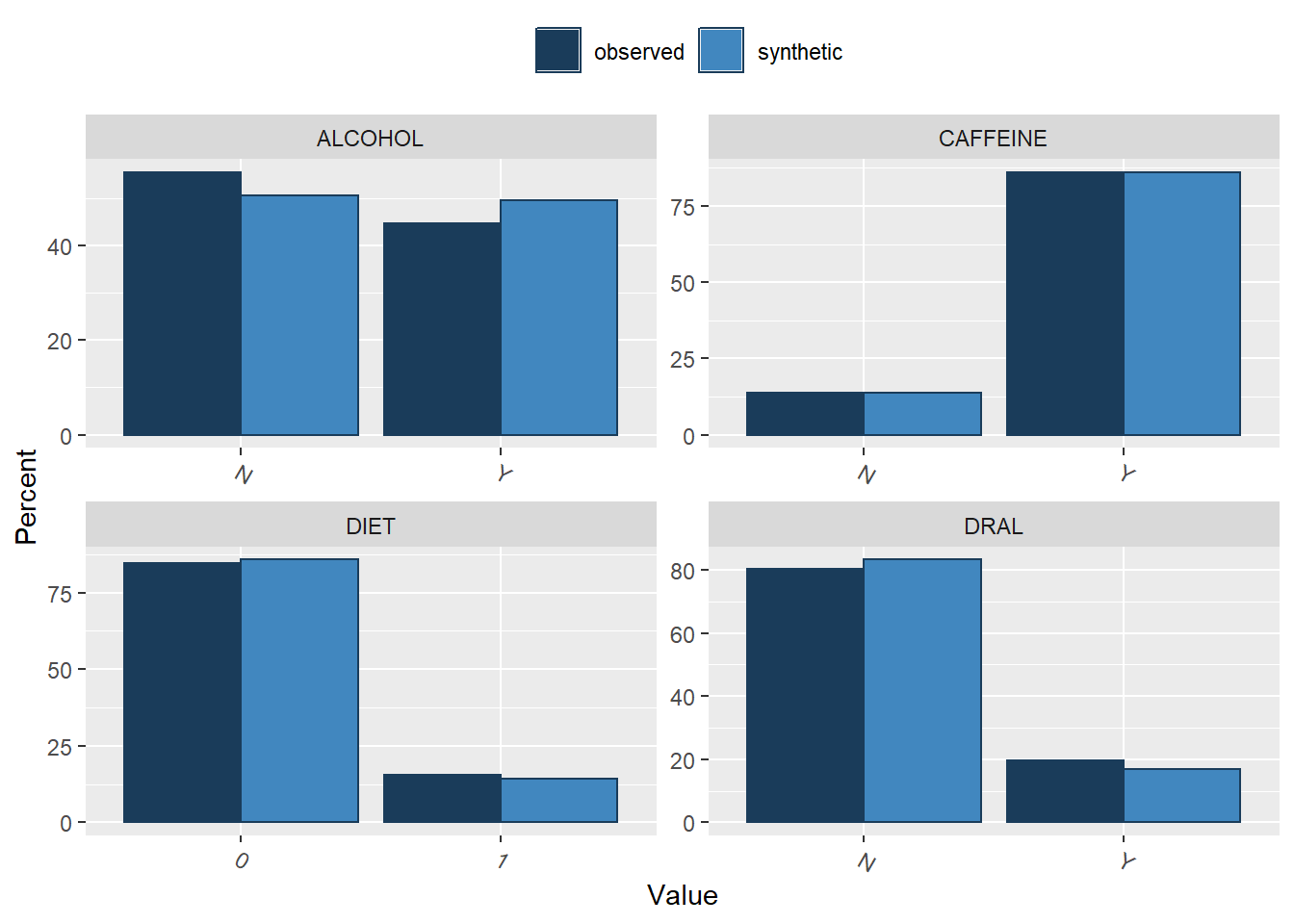 |
| 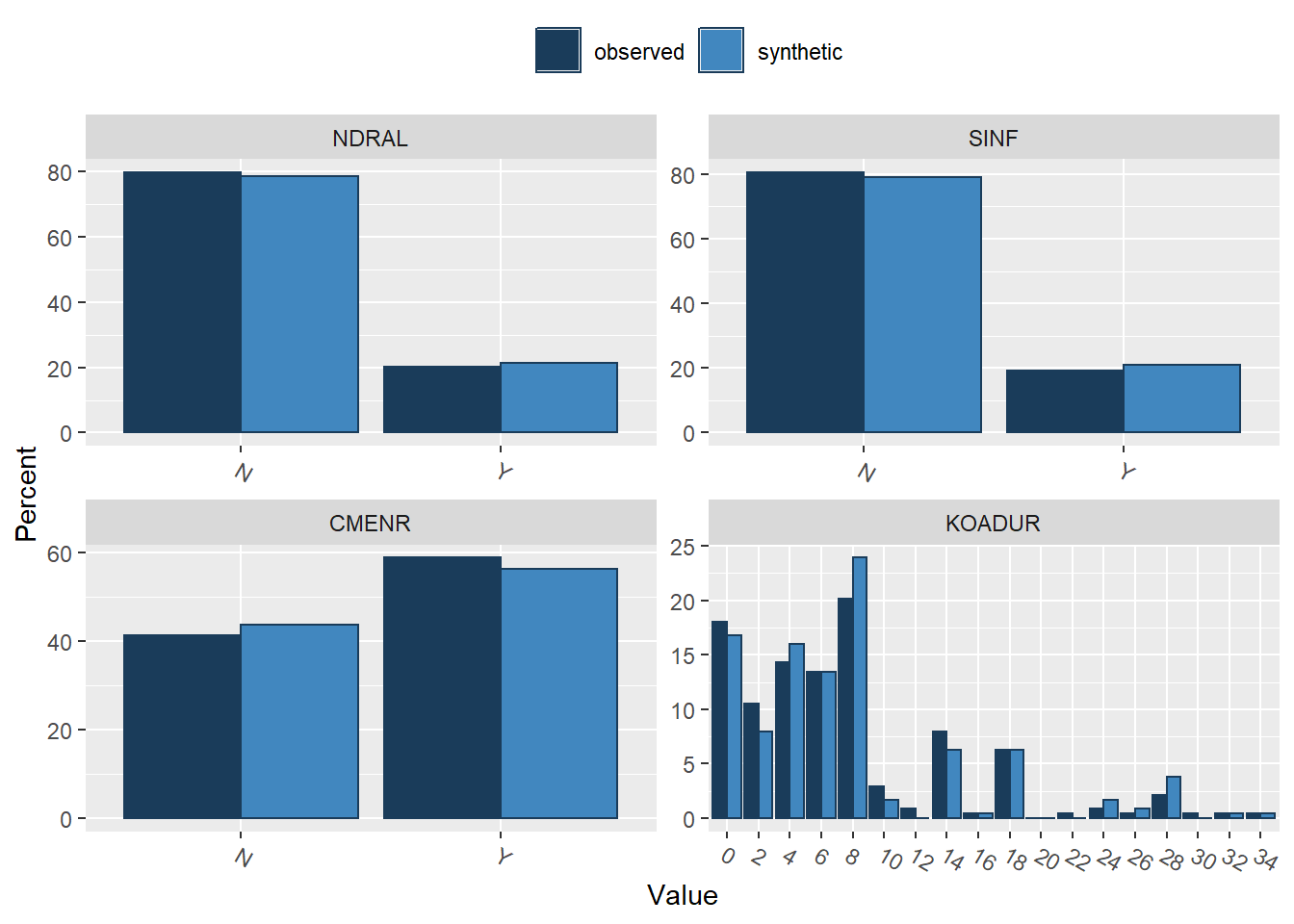 |
| 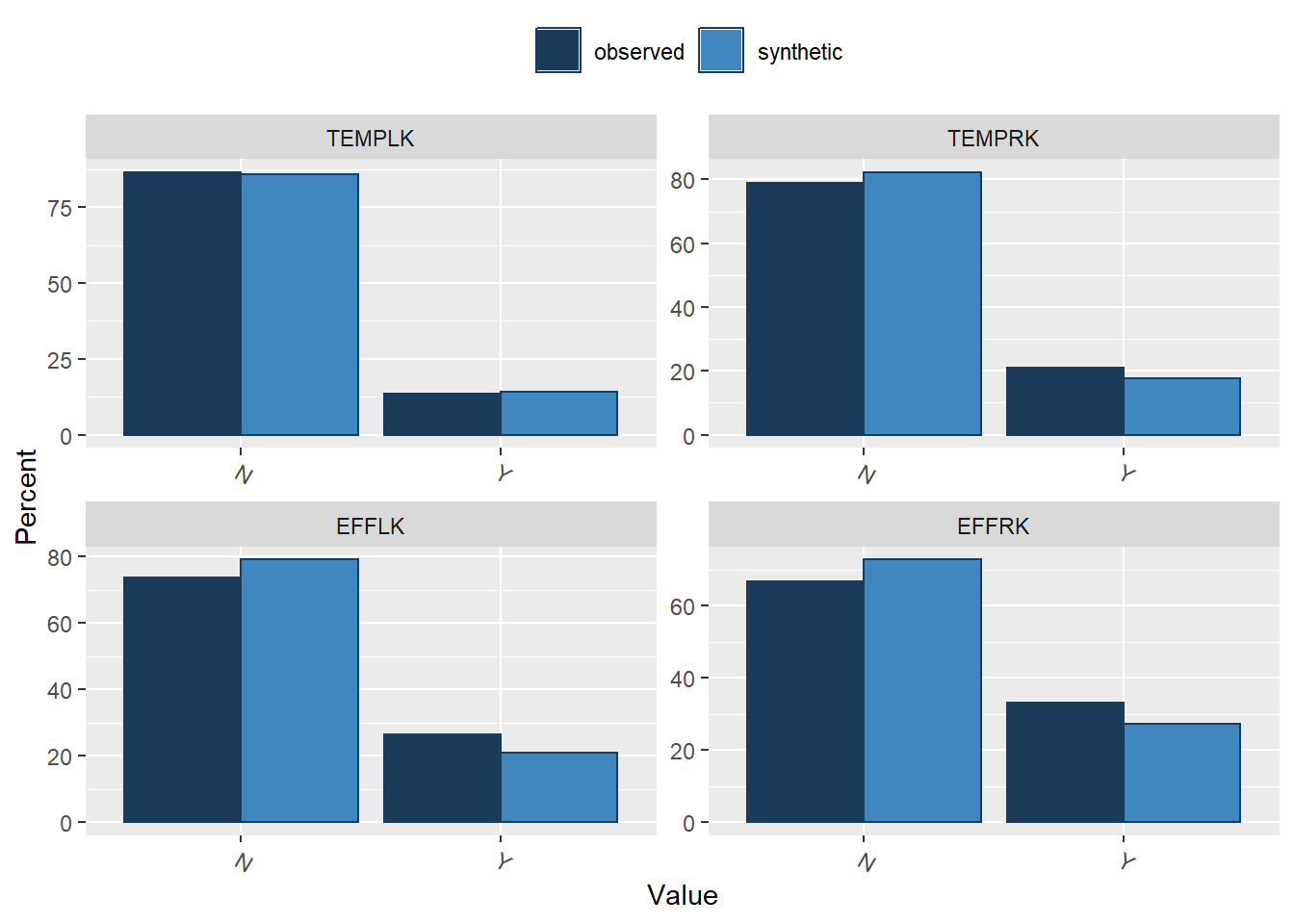 |
| 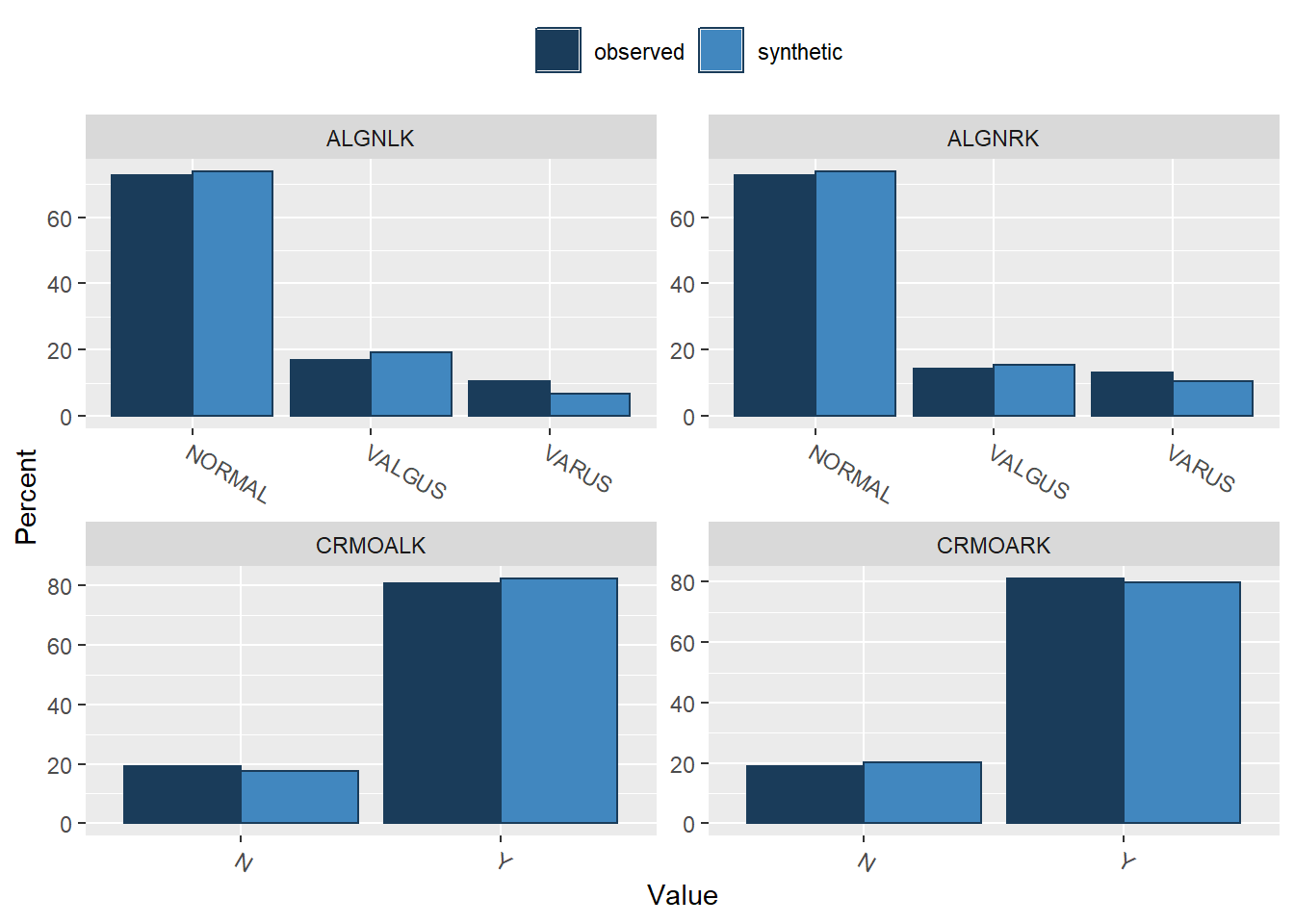 |
| 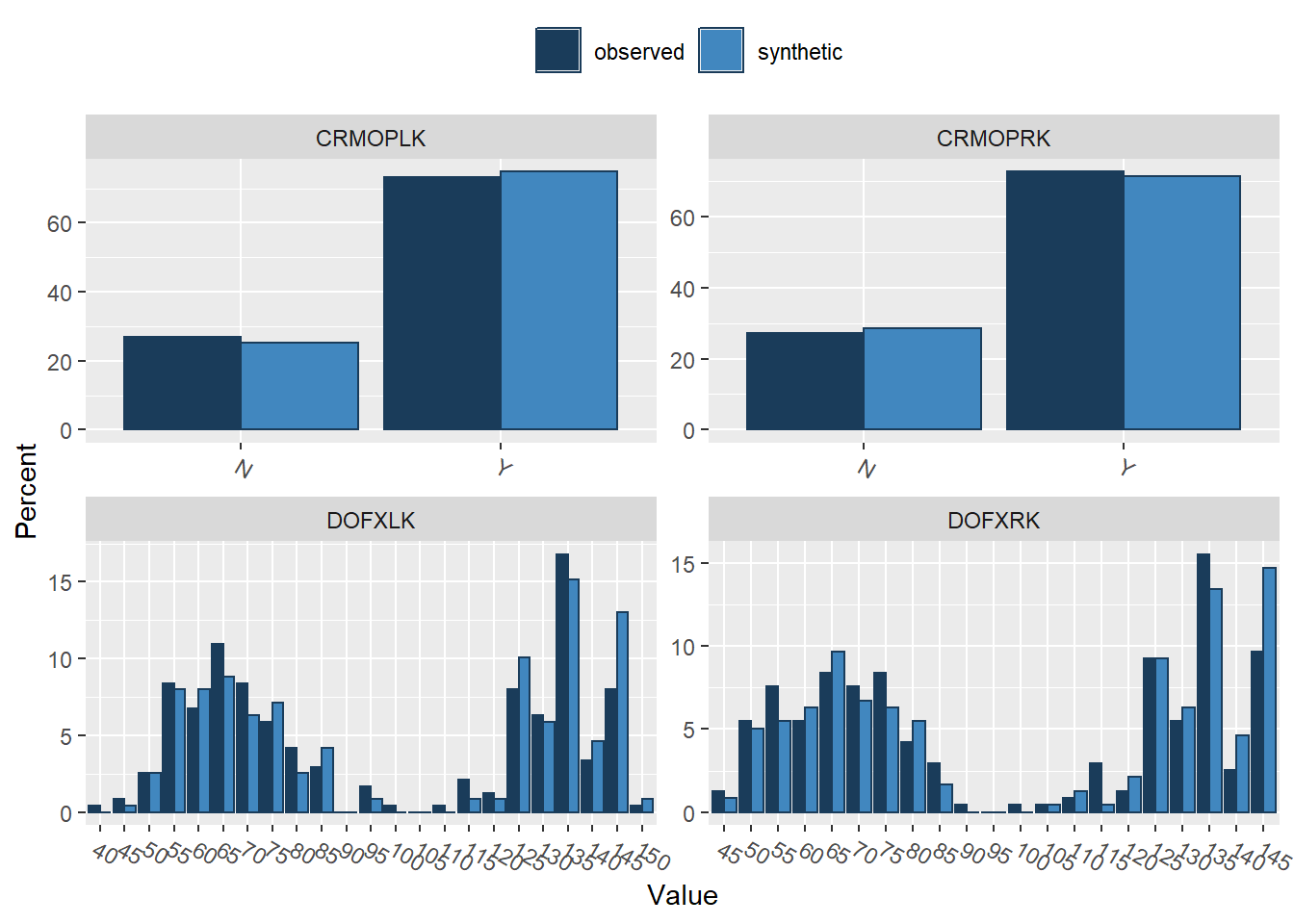 |

Figure S3 Comparison of variable distributions among the synthetic and observed data.

**Reference**

1. Polley EC, Van Der Laan MJ. Super learner in prediction.

2. Nowok B, Raab GM, Dibben C. synthpop: Bespoke Creation of Synthetic Data in R. *Journal of Statistical Software* 2016; 74: 1–26.
